# Antioxidant Effects, Antiproliferative Effects, and Molecular Docking of *Clinacanthus nutans* Leaf Extracts

**DOI:** 10.3390/molecules25092067

**Published:** 2020-04-29

**Authors:** Noor Zafirah Ismail, Zaleha Md Toha, Musthahimah Muhamad, Nik Nur Syazni Nik Mohamed Kamal, Nur Nadhirah Mohamad Zain, Hasni Arsad

**Affiliations:** Advanced Medical and Dental Institute, Universiti Sains Malaysia, Bertam, Kepala Batas 13200, Penang, Malaysia; piecesnzi@gmail.com (N.Z.I.); Zaleha.mdtoha@usm.my (Z.M.T.); musthahimahmuhamad@gmail.com (M.M.); niksyazni@usm.my (N.N.S.N.M.K.); nurnadhirah@usm.my (N.N.M.Z.)

**Keywords:** *Clinacanthus nutans*, antiproliferative effect, antioxidant, GC-MS, molecular docking

## Abstract

*Clinacanthus nutans* is a well-known herb that has been used as an alternative and therapeutic medicine, however more selective *C. nutans* extracts are needed. In this study, leaves were extracted with 80% methanol and further fractionated with *n*-hexane, dichloromethane, chloroform, n-butanol, and aqueous residue. Subsequently, the total phenolic content (TPC), total flavonoid content (TFC), antioxidant scavenging activity, and antiproliferative effects on breast cancer (Michigan Cancer Foundation-7 [MCF7]) and normal breast (Michigan Cancer Foundation-10A [MCF 10A]) cells of the extracts were measured. Additionally, molecular docking simulation of the major compounds from *C. nutans* extracts was conducted. The aqueous residue had the highest TPC and TFC, whereas the crude extract had the highest scavenging activity. Among the extracts, dichloromethane extract (CN-Dcm) was selected as it had the highest selectivity index (SI) (1.48). Then, the chosen extract (CN-Dcm) was proceed for further analysis. The compounds from CN-Dcm were identified using gas chromatography–mass spectrometry (GC-MS). The major compounds from CN-Dcm were further investigated through molecular docking studies. Palmitic acid and linolenyl alcohol were the compounds found in the CN-Dcm extract that exhibited the highest binding affinities with p53-binding protein Mdm-2. These results highlight the potential of *C. nutans* as a source of anticancer activities.

## 1. Introduction

Cancer is a major health problem and the most devastating life-threatening disease worldwide [1]. It is characterized by dysregulated cell growth, cell division, and cell death, and by the rapid proliferation of abnormal cells [2]. Global Cancer Incidence, Mortality and Prevalence (GLOBOCAN) reported that from 2012, 1.67 million new breast cancer cases occurred worldwide [3], and breast cancer is the most common cancer among Malaysian women [4]. Although the majority of breast cancer patients are female, a small number of men also suffer from breast cancer [4]. To date, chemotherapy is the first therapeutic strategy to fight cancers, despite the strong side effects. [5]. Natural products can assist the action of chemotherapy but cannot replace them yet. The demand for natural products, especially from medicinal plants, has increased because chemotherapeutic drugs are expensive, lead to morbidity, and have many side effects [6].

*Clinacanthus nutans* is a medicinal plant that has many medicinal properties, including treating lesions generated by the herpes-simplex virus, antiinflammatory, anticancer, antibacterial, and antivenom activities [7]. *C. nutans* is found in Malaysia, Vietnam, Indonesia, Thailand, and China [8,9], and occurs in most habitats [10]. Many studies carried out abroad have proven the effectiveness of *C. nutans* as an anticancer agent on several types of cancer cells. However, there is still lack of information on the effectiveness of *C. nutans* fraction solvent from various degrees of polarity on breast cancer cells. Michigan Cancer Foundation-7 (MCF7) is the favored cell line, mainly because of its sensitivity to the hormone estrogen, as it possesses estrogen receptors [11]. MCF7 is an ideal model for an in vitro hormone response study because it works well when incorporated into xenograft models, such as rabbit or mice models for in vivo tumorigenic experiments in the presence of estrogen, compared to breast cancer cell lines, such as MDA-MB-453 and SKBR3 cells, which do not have significant in vivo tumorigenic potential [12], or MDA-MB-231, which has poor in vivo metastatic effects [13]. Additionally, some MCF7 in vitro therapeutic response research studies have been translated to successful clinical trials and subsequent development of anticancer drugs [14].

Therefore, this study would be beneficial to further explore the antiproliferative effect of *C. nutans* extract on MCF7 cells. Additionally, many *C. nutans* studies did not evaluate the effect of *C. nutans* extracts on normal breast cancer cells. The use of normal cells ensures that the *C. nutans* extracts are not toxic to healthy cells. Thus, the goal of this study was to test the ability of different extracts of *C. nutans* to inhibit breast cancer cell proliferation. We measured the total phenolic content (TPC), total flavonoid content (TFC), and antioxidant scavenging activity of the *C. nutans* crude extract and its fractions using 2,2-diphenyl-1-picrylhydrazyl (DPPH) and 2,2′-azino-bis(3-ethylbenzothiazoline-6-sulfonic acid (ABTS) assays. We also evaluated the effects of the extracts on the cell growth of estrogen responsive breast cancer (MCF7) and normal breast (MCF 10A) cells, identified compounds from selected extracts using gas chromatography–mass spectrometry (GC-MS), and investigated the major compounds using molecular docking studies to evaluate their binding affinities with targeted apoptosis proteins.

## 2. Results and Discussion

### 2.1. Extraction Yield

Extraction of *C. nutans* leaves was performed using 80% methanol. The crude methanolic extract was further fractionated sequentially with different solvents (hexane, dichloromethane, chloroform, n-butanol, and aqueous residue). The effectiveness of plant extraction depends on the extraction method and on the solvent used, as solvents with different polarities have a significant effect on the chemical contents of the extracts. Thus, it is important to identify the optimal solvents for extraction of antioxidant compounds from medicinal plants [15]. Water was employed for the extraction because of its universal solubility of polar compounds, while methanol was chosen due to its ability to extract lower molecular weight polyphenols [16] and its tendency to yield relevant antioxidant and cytotoxic compounds from *C. nutans* [17]. Part of the crude methanolic extract was fractionated using different solvents to concentrate and enhance the purity of active compounds and remove unwanted interferences [16]. Table 1 shows the percentage yield of crude and fraction extracts of *C. nutans* leaves. The yield of the crude methanolic extract of *C. nutans* leaves (CN-Crd) was 6.85%, and the yield of the fractions varied from 2.18% to 37.71% in the following ascending order: aqueous residue (CN-Aqu) > *n*-butanol (CN-But) > dichloromethane (CN-Dcm) > *n*-hexane (CN-Hex) > chloroform (CN-Chl). The percentage yields were significantly different (*p* < 0.05).

### 2.2. TPC and TFC

**Figure 1:**
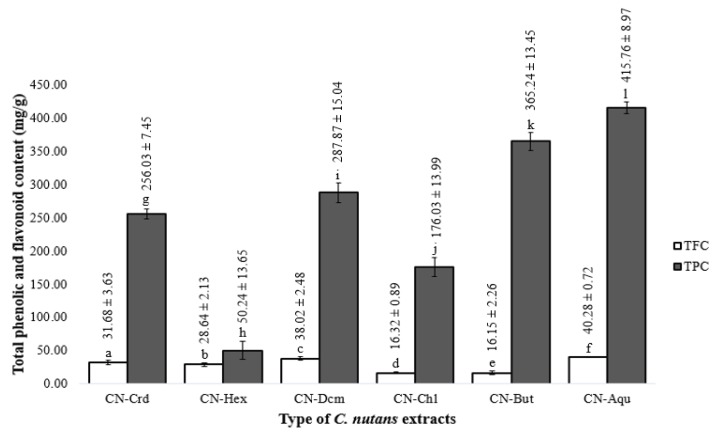
Total phenolic contents (TPC) and total flavonoid contents (TFC) of crude and fraction of *C. nutans* leaf extracts. A comparison of TPC and TFC in different *C. nutans* extracts; data represent mean ± SD, *n* = 3. LSD, least significant difference; level of significance: *p* < 0.05 using Bonferroni test analysis. Different lowercase characters represent significant difference.

The phenolic and flavonoid contents of the *C. nutans* extracts were measured because they are major contributors to the extract’s overall antioxidant activities. Figure 1 shows the TPC and TFC of the *C. nutans* extracts and fractions. The CN-Aqu fraction extract (415.76 mg gallic acid equivalent (GAE)/g extract) contained the highest amount of phenolics and the CN-Hex extract (50.24 mg GAE/g extract) contained the least. TPC values in descending order were as follows: CN-Aqu > CN-But > CN-Dcm > CN-Crd > CN-Chl > CN-Hex. The TPC of the CN-Aqu fraction was higher than other values reported in the literature for *C. nutans* extracts. For example, Sarega et al. [18] reported that the aqueous extract of *C. nutans* had a phenolic content of 63.77 ± 7.31 mg GAE/g extract. On the other hand, other plants reportedly have higher phenolic contents in the *n*-butanol extract, which agreed with the results of this study (i.e., CN-But had the second highest TPC). For instance, the phenolic content of the *n*-butanol extract of *Vernonia blumeoides* was 410 ± 0.8 mg GAE/g extract [19]. In our study, *n*-hexane extract of *C. nutans* had the lowest TPC; similarly, Johari et al. [20] reported that the hexane extract of *Pereskia bleo* had the lowest total phenolic content of 25.20 ± 0.01 mg GAE/g extract. The results of our analysis showed that TFC was lower than TPC in all extracts. This was not surprising, as flavonoids are subgroups of phenolics that contribute to the overall amounts of phenolics [20].

### 2.3. Antioxidant Capacities

The antioxidant capacities of the *C. nutans* extracts were quantified using 2,2-diphenyl-1-picrylhydrazyl (DPPH) and 2,2’-azino-bis(3-ethylbenzothiazoline-6-sulfonic acid (ABTS) assays. The use of at least two methods is recommended to evaluate and compare the antioxidant capacity of a sample [21]. Because they are stable radicals, DPPH and ABTS are widely used to determine the antioxidant activity of plants [22,23]. Table 2 shows the half-maximal effective concentration (EC_50_) of the extracts and fractions of *C. nutans* leaves. The EC_50_ is the concentration of antioxidant that causes a 50% decrease in radical absorbance, which is commonly assessed by measuring antioxidant concentration readings [24]. Trolox, which is a well-known natural antioxidant, was used as the standard. The *C. nutans* extracts ranged between 125 and 4000 µg/mL. The antioxidant activities measured using the ABTS assay were lower than values determined by the DPPH assay, which is in agreement with results reported by Lachman et al. [25]. Samples with lower EC_50_ values are considered to have high antioxidant capacity [26]. The EC_50_ values calculated from the DPPH and ABTS assays for the crude and fraction extracts of *C. nutans* ranged from 560.50 to 1530.00 µg/mL and from 476.30 to 1024.00 µg/mL, respectively. Based on the EC_50_ value, CN-Hex had the lowest antioxidant activity and CN-Crd had the highest antioxidant activity. Haron et al. [17] also reported that the crude methanol extract of *C. nutans* leaves showed the highest scavenging effect (DPPH = 21.18 mg Trolox/g, ABTS = 11.80 mg Trolox/g), while the hexane fraction had the lowest scavenging activity (DPPH = 1.06 mg Trolox/g). However, Alam et al. [27] reported that the crude methanol extract of *C. nutans* leaves had the lowest scavenging activity. These differences in results likely occurred because the plants are largely influenced by environmental conditions, such as temperature, rainfall, water variability, humidity, variations in soil pH, nutrient contents, and exposure to soil microorganisms [28]. Moreover, environmental factors interact with the genetics of the plants, which lead to genetic variations [29] that affect the phytochemical contents.

### 2.4. Antiproliferative Assay

The antiproliferative activity of the extracts was assessed using the sulforhodamine B (SRB) assay. SRB is a fluorescent dye used to quantify the proteins present in cultured cells. The SRB dye binds to the amino acids of cellular proteins and then the dye can be used to estimate the cell viability [30]. Based on the SRB assay, we compared the results of the SRB assay with 3-(4,5-dimethylthiazol-2-yl)-5-(3-carboxymethoxyphenyl)-2-(4-sulfophenyl)-2*H*-tetrazolium (MTS) and alamar blue assays at 72 h of exposure (Table S1). The results showed no significant differences between three cytotoxicity assays (*p* > 0.05), which were in agreement with studies reported by Aslantürk [31] and Vajrabhaya and Korsuwannawong [32]. The 3-(4,5-dimethylthiazol-2-yl)-2,5-diphenyl-2*H*-tetrazolium bromide (MTT) assay was a commonly used cytotoxicity assay. However, van Tonder et al. [33] describes that the MTT assay was less accurate in detecting changes in cell numbers. They [33] also expressed that that SRB assay appears to be more sensitive than MTT assay, which was in line with the studies by Skehan et al. [34] and Vajrabhaya and Korsuwannawong [32], who reported that SRB assay had better linearity with cell number and higher reproducibility. Thus, we choose SRB assay to report the antiproliferative effects of crude and fraction extracts of *C. nutans*.

**Figure 2:**
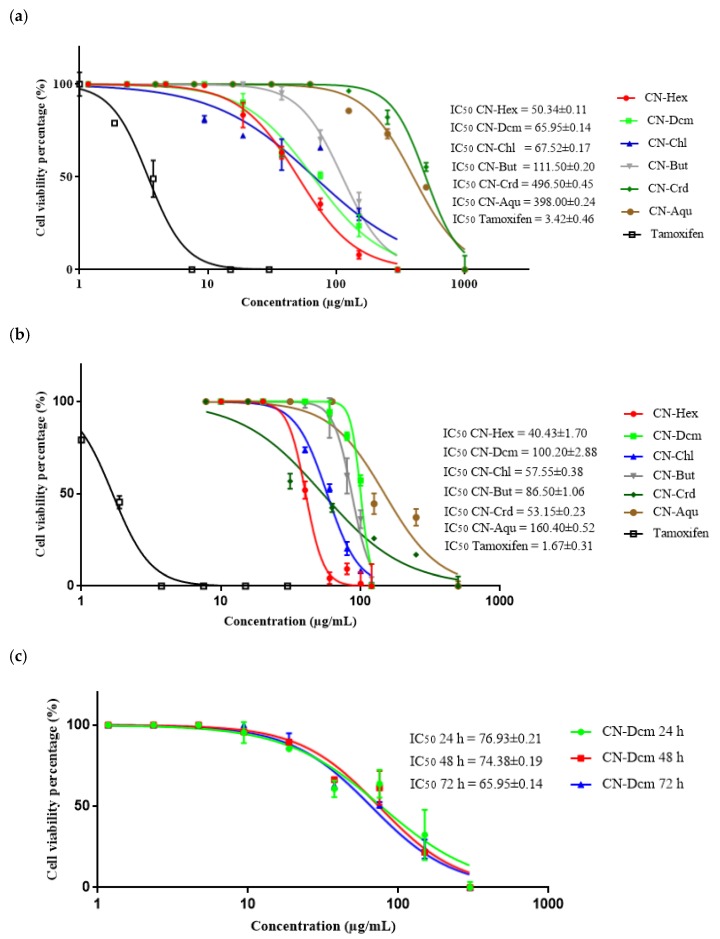
Preliminary screening of the IC_50_ of *C. nutans* crude and fraction extracts using the sulforhodamine B (SRB) assay: (**a**) antiproliferative effect of *C. nutans* extracts on MCF7 cell viability; (**b**) antiproliferative effect of *C. nutans* extracts on MCF 10A cells; (**c**) antiproliferative of the CN-Dcm fraction extract on MCF7 cells at 24, 48, and 72 h of exposure. Results are presented as mean ± standard deviation (SD) (*n* = 3). CN-Crd, crude methanolic extract; CN-Hex, hexane fraction extract; CN-Dcm, dichloromethane fraction extract; CN-Chl, chloroform fraction extract; CN-But, *n*-butanol fraction extract; CN-Aqu, aqueous residue fraction extract.

The antiproliferative activities of the crude and fraction extracts of *C. nutans* leaves were tested on MCF7 and MCF 10A cells to determine their inhibitory effects on cell proliferation. Figure 2 shows the effect of various concentrations of crude and fraction extracts on cell viability of MCF7 and MCF 10A cells. Tamoxifen was used as the positive control and its IC_50_ for MCF7 cells was 3.42 ± 0.46 µg/mL. Tamoxifen also inhibited MCF 10A normal breast cells, with an IC_50_ value of 1.67 ± 0.31 µg/mL. The CN-Crd extract inhibited 50% of MCF7 cell growth at 496.50 ± 0.45 µg/mL. This result is in agreement with the observed cytotoxic effect of the crude methanol extract of *C. nutans* leaves on human immortalized myelogenous leukemia (K-562) cells, with IC_50_ >100 µg/mL at 72 h [35]. Our results showed that CN-Crd had a low inhibitory effect on MCF7 cells. Haron et al. [36] also showed that the crude extract had a low inhibitory effect on cervical cancer (HeLa) cells. Based on the antiproliferative activity of MCF10A, the results demonstrated that CN-Crd of *C. nutans* leaves inhibited the growth of MCF 10A cells, with an IC_50_ of 53.15 ± 0.23 µg/mL.

The CN-Crd extract was sequentially fractionated using *n*-hexane, dichloromethane, chloroform, and *n*-butanol, and the antiproliferative effects of these fractions on MCF7 cells were investigated. Fractionation was performed because fractions of crude alcoholic extracts may have high inhibitory effects against cancer cells [36,37]. Adebayo et al. [37] found that the hexane fraction of the crude 80% ethanolic extract of *Moringa oleifera* had stronger antiproliferation activity against cancer cells compared to the crude extract. Similarly, hexane, dichloromethane, ethyl acetate, and butanol fractions of the crude methanolic extract of *Ziziphus mauritiana* bark had high inhibitory effects on MCF7, HeLa, prostate cancer (PC3), and lung cancer (NCI-H460) cells [38].

In our study, the CN-Hex fraction had the greatest inhibitory effects on MCF7 cells, with an IC_50_ of 50.34 ± 0.11 µg/mL, followed by the CN-Dcm fraction (IC_50_ = 65.95 ± 0.17 µg/mL), CN-Chl (IC_50_ = 67.52 ± 0.17 µg/mL), CN-But (IC_50_ = 111.50 ± 0.20 µg/mL), and CN-Aqu (IC_50_ = 398.00 ± 0.24 µg/mL). Wang et al. [39] reported that the IC_50_ of the hexane fraction of *C. nutans* leaves was 84.77 ± 3.43 µg/mL, which was higher than the IC_50_ for CN-Hex; this means that the CN-Hex fraction was more toxic than the hexane fraction in Wang et al.’s study [39]. There are no published reports about the antiproliferative effects of the dichloromethane, chloroform, and *n*-butanol fractions of the crude methanolic extract of *C. nutans* on MCF7 cells. However, the inhibitory effect of the CN-Dcm fraction on the growth of MCF7 cells in our study was comparable to the observed antiproliferative effect of the dichloromethane fraction of *C. nutans* leaves on HeLa cells that was reported by Haron et al. [36]. CN-Dcm was more effective for MCF7 cells because it had a lower IC_50._ On the other hand, CN-Chl inhibited MCF7 cell growth at a lower concentration (67.52 ± 0.17 µg/mL). There was no inhibition of MCF7 cell growth when the cells were treated with CN-Crd and CN-Aqu extracts at concentrations below 100 μg/mL.

The fraction extracts were also tested for their effects on MCF 10A cells (Figure 2b). MCF 10A cells were used to obtain the selective indices (SI) of the extracts. The SI value was calculated as the ratio of the IC_50_ values of the extracts on MCF 10A cells relative to those in the MCF7 cells. According to Segun et al. [40], an SI value > 1 one suggests that an extract is less toxic to normal cells compared with cancer cells, thus compounds with high SI values can be assumed to offer potential safer therapy. Table 3 shows the SI values of the crude and fraction extracts of *C. nutans* leaves. In ascending order, the SI values were as follows: CN-Dcm > CN-Chl > CN-Hex > CN-But > CN-Aqu > CN-Crd. CN-Dcm inhibited MCF 10A cell growth, with an IC_50_ value of 100.20 ± 2.88 µg/mL and an SI value of 1.48, which means that this fraction extract was selective. On the other hand, CN-Hex, CN-Chl, CN-Aqu, and CN-Crd inhibited MCF 10A cell growth with IC_50_ values of 40.43 ± 1.70 µg/mL, 57.55 ± 0.38 µg/mL, 160.40 ± 0.52 µg/mL, and 53.15 ± 0.23 µg/mL, respectively, and had lower SI values than the CN-Dcm fraction. These results suggest that these fractions were more toxic to the normal cells than to the cancer cells. The growth of MCF 10A cells exposed to CN-But was inhibited at an IC_50_ value of 86.50 ± 1.06 µg/mL, whereas it inhibited the growth of MCF7 cancer cells with an IC_50_ value of 111.50 ± 0.20 µg/mL. Thus, the CN-But fraction had selective toxicity to the cancer cells, unlike CN-Hex, CN-Chl, CN-Aqu, and CN-Crd, which non-selectively inhibited the proliferation of both MCF7 and MCF 10A cells.

Based on these results, CN-Dcm was the most selective fraction, as it selectively inhibited MCF7 cells and was less toxic to MCF 10A cells. Therefore, it was selected for further investigation. The results were in agreement with previous *C. nutans* studies, in which non-polar to semipolar extracts had higher antiproliferative activity than polar extracts [35,41,42,43]. However, none of the crude and fraction extracts of *C. nutans* leaves followed the criteria of the National Cancer Institute, which requires a crude plant extract to have an IC_50_ < 30 μg/mL for preliminary assay [44]. The cell viability assay was repeated using the CN-Dcm fraction extract for different incubation times (24, 48, and 72 h). The IC_50_ values for the three time points were plotted (Figure 2c) and show that at 24 h of treatment, CN-Dcm inhibited 50% of cell proliferation at a concentration of 76.93 ± 0.21 µg/mL, while at 48 h the IC_50_ of the extract was 74.38 ± 0.19 µg/mL and at 72 h it was 65.95 ± 0.14 µg/mL. Thus, the inhibitory activity of this extract occurred in a dose- and time-dependent manner. The IC_50_ of the CN-Dcm fraction decreased when the period of incubation increased.

### 2.5. GC-MS Spectral Analysis of Compounds Presents in CN-Dcm

We were able to separate and identify various constituents of CN-Dcm using GC-MS analysis. Table 4 summarizes the 14 components identified in the CN-Dcm fraction with their molecular formula, peak percentage, and similarity index. Compounds were identified by referring to the corresponding compound in the National Institute of Standards and Technology (NIST) library; a similarity index of at least 80% was required for identification. The GC-MS analysis revealed the presence of two major components: linolenyl alcohol (29.10%) and palmitic acid (23.84%).

### 2.6. Molecular Docking

#### 2.6.1. Drug-Likeness and Toxicity Prediction

**Figure 3:**
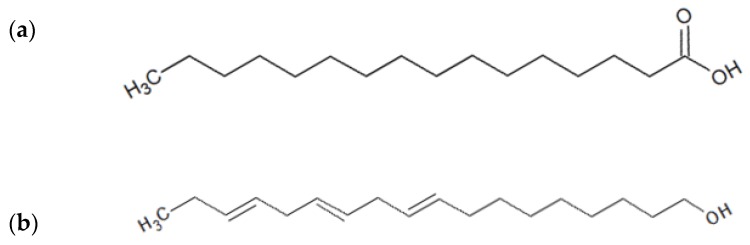
The major compounds found in CN-Dcm fraction extract of *C. nutans* leaves: (**a**) palmitic acid; (**b**) linolenyl alcohol.

The two major compounds found in the CN-Dcm fraction, palmitic acid and linolenyl alcohol (Figure 3), were subjected to drug-likeness and toxicity prediction using Lipinski’s rule of five; this rule determines the consistency of orally active drugs [45]. Lipinski’s rule of five [45] states that a drug molecule generally does not violate more than one of the following five rules: molecular mass < 500 Da, high lipophilicity (expressed as LogP < than 5), < 5 hydrogen bond donors, < 10 hydrogen bond acceptors, and molar refractivity between 40 and 130 [46,47]. As displayed in Table 5, linolenyl alcohol and palmitic acid did not violate any rules, suggesting that they could be suitable for oral administration.

#### 2.6.2. Molecular Docking Analysis

Molecular docking analysis is widely used in drug discovery to understand the drug–receptor interaction and gene pathway [48]. The antiproliferative effect of the *C. nutans* extracts may be caused by activation of apoptosis proteins. Apoptosis, or programmed cell death, is an important component of regulation of cell growth and maintenance of tissue homeostasis, and it is restricted during uncontrolled growth of damaged cells [49]. Apoptosis is also the hallmark of cancer [50]. In this analysis, we studied the following apoptosis-related proteins: tumor necrosis factor-α (TNF-α), p53-binding protein Mdm-2, and caspase-3. The p53 tumor suppressor protein activates transcription of proapoptotic genes that encode members of the Bcl-2 family, as well as TNF-related apoptosis [51]. Activation of p53 only occurs through its dissociation from its inhibitor, MDM2; thus, MDM2 inhibitors are considered to be proapoptotic agents [52,53]. Álvarez et al. [54] reported that the cytokinine TNF-α can promote activation of caspase-3 by expresssing Fas ligand (FasL) through nuclear factor of activated T-cells (NFAT) activation in neuroblastoma cells. Caspase-3 is an essential marker that has been shown to be an entry point in the apoptotic signalling pathway. Hence, caspase-3 is important for the formation of apoptoic bodies and dismantling of the cells, and may function before or after the loss of cell viability [55].

In this study, the proteins TNF-α, p53 binding protein Mdm-2, and caspase-3 were docked with palmitic acid and linolenyl alcohol. Table 6 shows the best result for each compound and the apoptosis proteins. Palmitic acid and linolenyl alcohol had the highest binding affinities towards p53-binding protein Mdm-2, with binding energy values of −4.22 kcal/mol and −4.56 kcal/mol, respectively. According to Pantsar and Poso [56], the lowest binding energies of ligands towards the targeted proteins result in the highest binding affinities. The lowest binding affinities detected were −2.45 kcal/mol and −2.99 kcal/mol for palmitic acid and linolenyl alcohol with TNF-α protein, respectively.

**Figure 4:**
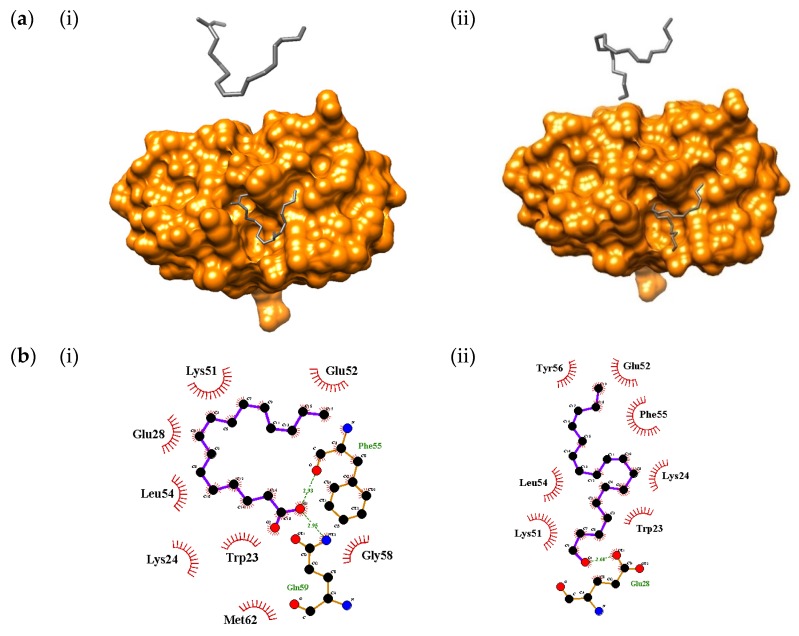

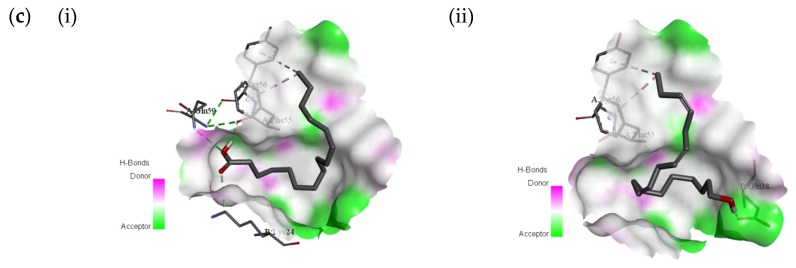
Molecular docking and interaction pattern analysis of the compounds with apoptosis proteins: (**a**) the best binding affinities of the compounds with targeted proteins; (**b**) interaction of the compounds and active site residues using LigPlot+; (**c**) interaction of the compounds with active site residues using Discovery Studio Visualizer 4.1 client. The compounds and proteins correspond to (i) palmitic acid with p53-binding protein Mdm-2 and (ii) linolenyl alcohol with p53-binding protein Mdm-2.

Protein docking analysis showed that linolenyl alcohol had better binding affinity towards p53-binding protein Mdm-2 compared to palmitic acid. Figure 4 shows results of the interaction pattern analysis of the compounds with apoptosis proteins. Based on the result from Discovery Studio Visualizer 4.1 client, the molecular interactions of linolenyl alcohol with p53-binding protein Mdm-2 formed two Pi-Alkyl interactions with Phe-55 and Tyr-56, two hydrogen bonds were formed, and two hydrophobic interactions occurred. Palmitic acid formed eight hydrogen bonds with four residues, namely Gln-59, Lys-24, Phe-55, and Tyr-56. Palmitic acid also formed two Pi-Alkyl interactions with Phe-55 and Tyr-56 residues. The finding demonstrated that caspase-3 bound to palmitic acid and linolenyl alcohol, with binding energies of −3.56 kcal/mol and −3.93 kcal/mol, respectively. Mutazah et al. [57] previously reported that entadamide C (1) and clinamide D (2) from *C. nutans* extracts bound favorably to the caspase-3 binding site, with binding energies of −4.28 kcal/mol and −4.84 kcal/mol, respectively. Thus, these results showed that such interactions of *C. nutans* compounds are important for the activation of apoptosis-related proteins.

## 3. Materials and Methods

### 3.1. Reagents

Analytical grade methanol, hexane, dichloromethane, chloroform, *n*-butanol, dimethylsulfoxide (DMSO), acetic acid, and HPLC-grade methanol were purchased from Qrec (Asia) Sdn. Bhd (Rawang, Selangor, Malaysia). Folin–Ciocalteu reagent, sodium carbonate, DPPH, ABTS, aluminum chloride, potassium persulfate, sulforhodamine B, tris buffer, and trichloroacetic acid were purchased from Sigma-Aldrich (St. Louis, MO, USA). The standards for antioxidant assays (quercetin, gallic acid, and vitamin E analogue 6-hydroxy 2,5,7,8-tetramethylchroman-2-carboxylic acid (Trolox)) were purchased from Sigma-Aldrich (St. Louis, MO, USA). Roswell Park Memorial Institute culture medium (RPMI-1640) and fetal bovine serum were purchased from Nacalai Tesque (Kyoto, Japan). Dulbecco’s modified Eagle medium (DMEM), penicillin-streptomycin (PenStrep), horse serum, epidermal growth factor (EGF), hydrocortisone, and insulin were purchased from Gibco (Paisley, UK). Trypan blue was purchased from Hycel de México (Zapopan, México).

### 3.2. Plant Material and Sample Preparation

Fresh samples of *C. nutans* were purchased from HERBagus at Kepala Batas, Penang, Malaysia (5.5185° N, 100.4799° E), and the plant was identified using DNA barcoding markers [9]. The leaves were separated from the plants and washed with distilled water. Fresh leaves (3000 g) were ground and soaked with 80% methanol (2.5 L) at room temperature (20 °C). The mixtures were continuously shaken at 130 rpm for 24 h on a shaker. The filtrate was collected by filtration of the mixtures through a Whatman No. 1 filter paper by gravity. The extraction was repeated two more times and the crude extracts were pooled together. Methanol was evaporated from the pooled crude extract using a rotary evaporator (125 hpa, 40 °C) and the crude extract was freeze-dried.

**Figure 5:**
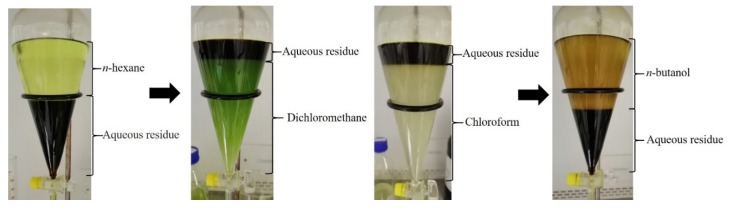
Fractionation of crude extracts using four solvents of different polarities (*n*-hexane, dichloromethane, chloroform, and n-butanol, along with aqueous residue).

Next, 70 g of crude extract were dissolved with distilled water (100 mL) and subjected to liquid–liquid fractionation with each of the following four solvents (200 mL) of different polarities: *n*-hexane (CN-Hex), dichloromethane (Cn-Dcm), chloroform (CN-Chl), and *n*-butanol (CN-But) (Figure 5). The mixtures were mixed vigorously with the solvents (200 mL) in a separatory funnel for 30 min. The extraction solvents were evaporated from the fractions, and water was also evaporated. All samples were kept at 4 °C until further use.

### 3.3. Determination of Antioxidant Activities

#### 3.3.1. TPC

The TPC of *C. nutans* crude and fraction extracts was measured following Ismail et al. [28], with slight modification. First, 1000 µg of each extract were diluted using 50% methanol to obtain 1000 µg/mL of sample concentration and then 1 mL of 100% Folin–Ciocalteu reagent was mixed with 9 mL of distilled water. Next, 0.3 g of sodium carbonate was dissolved in 4 mL of distilled water to obtain 7.5% sodium carbonate. The extracts (20 µL) were added to 96-well plates and mixed with 100 µL of 10% Folin–Ciocalteu reagent. The mixtures were incubated for 5 min in the dark. Next, 80 µL of 7.5% sodium carbonate were added to the mixtures, which were incubated for 30 min before reading the absorbance at 760 nm using a spectrophotometer (OMEGA BMG Labtech, Ortenberg, Germany). The analysis was performed in triplicate. A total of nine concentrations of gallic acid ranging from 3.9 to 1000 µg/mL were made by serial dilution. The same procedure was repeated for the gallic acid concentrations and the calibration line was created. The TPC was expressed as percentage of total gallic acid equivalents per g extract (mg GAE/g). The TPC regression curve for the gallic acid standard had the equation y = 0.0076x − 0.0031, with an R^2^ value of 0.9999.

#### 3.3.2. TFC

The TFC of *C. nutans* crude and fraction extracts was measured following Ismail et al. [28]. A 100 µL aliquot of the prepared solutions (1000 µg/mL) of crude and fraction extracts of *C. nutans* leaves was added to 100 µL of 2% aluminum chloride and incubated for 10 min in the dark. The absorbance of the mixture then was read using a spectrophotometer at 420 nm. The analysis was performed in triplicate. Serial dilutions of the standard quercetin starting from 1000 µg/mL were used to generate a standard curve. The TFC was expressed as percentage of total quercetin equivalents per g extract (mg QE/g). The TFC regression curve for the quercetin standard had the equation y = 0.0107x + 0.0272, with an R^2^ value of 0.9989.

#### 3.3.3. DPPH

The antioxidant capacity was measured using the DPPH radical scavenging method, as described by Ismail et al. [28], with some modifications. A 0.6 mM DPPH stock solution was prepared by dissolving 6 mg of DPPH in 25 mL of methanol. The DPPH working solution was obtained by dissolving the DPPH stock solution in methanol until the absorbance reading at 517 nm was 1.1 ± 0.02 nm. Next, 50 µL of the *C. nutans* crude and fraction extracts ranging from concentrations of 125 to 4000 µg/mL were added to 100 µL of DPPH working solution in 96-well plates. The mixtures were incubated at 30 min in the dark. The absorbance was read at 517 nm using the spectrophotometer. Trolox ranging from 3.9 to 1000 µg/mL was used as a positive control. The experiment was performed in triplicate for the standard and the extracts. The inhibition ratio was calculated as percentage of inhibition using the following formula: percentage inhibition (%) = ((absorbance of control) − (absorbance of test sample)/absorbance of control) × 100%. The extract concentration providing the half-maximal effective concentration (EC_50_) was calculated using a graph by plotting the percentage effective concentration against extract concentration. The data were presented as mean values ± standard deviation (SD).

#### 3.3.4. ABTS

The ABTS radical cation decolorization assay described by Fidrianny et al. [58] was used. The ABTS radical cation working solution was produced by reacting 7.5 mM ABTS stock solution with 3.8 mM potassium persulfate, and the mixture was allowed to stand in the dark at room temperature for 16 h before use to yield a dark-colored solution containing ABTS•+ radicals. The ABTS radical cation working solution was then diluted with methanol for an initial absorbance of about 0.70 ± 0.02 at 734 nm. Mixtures in a total volume of 100 µL containing 90 µL of ABTS radical cation working solution and 10 µL of varying concentrations of the extract (125–4000 µg/mL) were incubated in the dark. Appropriate solvent blanks were run with each assay. The absorbance was read by the spectrophotometer at 734 nm and compared with the Trolox control (3.9–1000 µg/mL). The assay was performed in triplicate. The scavenging activity was estimated based on the EC_50_ of ABTS radicals scavenged. The data were presented as mean values ± SD.

#### 3.3.5. Statistical Analysis

All the experiments for determination of TPC, TFC, and antioxidant properties using DPPH and ABTS were conducted in triplicates. The values are expressed as the mean ± standard deviation (SD). The statistical analysis of the results was done by IBM Statistical Package for Social Sciences (SPSS) Statistical Version 24 for statistical computing. Analysis of variance (ANOVA) and significance of differences among means were tested by one-way ANOVA and least significant difference (LSD) on mean values, respectively.

### 3.4. Cell Culture for Cytotoxicity Testing

Cryovials containing MCF7 and MCF 10A cells were thawed in a water bath at 37 °C. The cells were then transferred into a 15 mL centrifuge tube and centrifuged at 1000 rpm for 5 min to remove the cryopreservative agent (DMSO). The MCF7 cells were introduced into a T-25 falcon flask and cultivated in RPMI-1640 medium supplemented with 10% (*v*/*v*) fetal bovine serum and 1% PenStrep to increase and stimulate cells survival and proliferation. PenStrep was added to preferentially kill any bacteria present that might contaminate the cells. The MCF 10A were cultivated in DMEM supplemented with 5% horse serum, 20 mg/mL epidermal growth factor, 0.5 mg/mL hydrocortisone, 10 µg/mL insulin, and 5 mL of PenStrep. Both cell types were incubated at 37 °C in a CO_2_ incubator supplemented with 5% CO_2_. The media were replaced every 3 days until the cells were confluent and ready to be sub-cultivated. The medium in each flask was discarded when the cells reached confluence. The cells were then washed two times with 2 mL of phosphate-buffered saline to ensure removal of any residue of spent culture medium and dead cells. Cells were detached by adding 200 μL of trypsin and incubated for 5 min. The process of trypsinization was enhanced by gently tapping the flask a few times. Once the cells appeared rounded and single under the microscope, 4 mL of medium were added to the cells to inactivate the trypsin. The cell suspension was transferred to a 15 mL falcon tube and centrifuged at 1000 rpm for 5 min. Cells then were resuspended in the culture media.

#### 3.4.1. Cell Viability Assay

The effects of *C. nutans* crude extract and its fractions on the viability of MCF7 and MCF 10A cells were determined using the sulforhodamine B (SRB) assay, as described by Skehan et al. [34], with some modifications. Concentrations of MCF7 and MCF 10A cells growing in the exponential phase (1 × 10^4^ cells/mL) were produced using an automated cell counter. Aliquots of 100 μL of medium were seeded into each well of 96-well plates and incubated in a CO_2_ incubator for 24 h. The CN-Crd extract and CN-Aqu residue extract were dissolved in both complete media, whereas CN-Hex, CN-Dcm, CN-Chl, and CN-But fractions were dissolved in DMSO. The working solutions of fraction extracts were in 0.1% DMSO. This was to make sure that the highest DMSO concentration in the cell culture was within the acceptable limit (0.1–0.5%) [59]. Briefly, the cells were incubated in 96-well plates with a serial dilution of extracts, starting with 1000 μg/mL (CN-Crd and CN-Aqu) and 300 μg/mL (CN-Hex, CN-Dcm, CN-Chl, and CN-But fraction extracts) for 72 h at 37 °C with 5% CO_2_.

For MCF 10A cells, the new range of crude and fraction extracts of *C. nutans* leaves was made based on the half-maximal inhibitory concentration (IC_50_) of MCF7 cancer cells. The new range for MCF 10A cells started with 500 μg/mL (CN-Crd and CN-Aqu) and 120 μg/mL (CN-Hex, CN-Dcm, CN-Chl, and CN-But fraction extracts). The positive control experiment involved tamoxifen, and the negative control was untreated media or 0.1% DMSO. At the end of the incubation period, the cells were fixed with 50 µL of 50% cold trichloroacetic acid for 30 min at room temperature, followed by gentle tap water washing (4x) and drying. The cells then were then stained with 100 μL of 0.4% SRB in 1% acetic acid for 30 min, followed by washing with 1% acetic acid (4x). The plate was dried, 100 μL of 10 mM Tris buffer were added to each well, and the plate was shaken for 5 min. Relative cell viability was measured by scanning at 570 nm on a microplate reader (OMEGA BMG Labtech, Subang Jaya, Selangor, Malaysia). The cytotoxicity was determined using the previously described formula reported by Bendale et al. [60]:

Cytotoxicity (%) = Optical density of (sample − blank)/Optical density of (control − blank) × 100%(1)

#### 3.4.2. Selective Index (SI)

The SI was used to determine the cytotoxic selectivity of the substances tested. It was calculated according to the following equation [61]:

SI = IC_50_ (normal cells)/IC_50_ (cancer cells) (2)

The extract with highest SI CN-Dcm) was selected for another cell viability assay at different incubation times (24, 48, and 72 h).

### 3.5. GC-MS

For the GC-MS analysis, 1000 µg/mL of CN-Dcm was dissolved in 1 mL of HPLC grade methanol. The secondary metabolites from CN-Dcm were determined using an Agilent gas chromatograph model 6890 equipped with an Agilent 19091S-433 capillary column, (5%-phenyl)-methylpolysiloxane phase (HP-5MS) 0.25 mm × 30 m × 0.25µm) (Santa Clara, CA, USA). Helium gas was used as the carrier gas at 1.0 mL per minute with split mode injection. The oven temperature was set as follows: 70 °C was held for 2 min, then it was increased to 280 °C at a rate of 20 °C per min for 20 min. The total run time was 32.50 min.

### 3.6. Molecular Docking

#### 3.6.1. Drug-Likeness and Toxicity Predictions

Lipinski’s rule of five was used to predict the drug-likeness; this rule determines the consistency of orally active drugs [45,47]. In this study, the selected compounds (palmitic acid linolenyl alcohol) were screened using the SwissADME web tool predictor [62]. The SwissADME predictor provides data on the numbers of hydrogen acceptors, hydrogen donors, and rotatable bonds.

#### 3.6.2. Protein Model and Compound Structure

Protein structures of tumor necrosis factor alpha (TNF-α, PDB ID: 2AZ5), p53-binding protein Mdm-2 (PDB ID: 1YCR), and caspase-3 (PDB ID: 6CKZ) were retrieved from the Research Collaboratory Structural Bioinformatics Protein Data Bank. All data files were saved in .pdb file format. Water and ligand molecules from the proteins were removed using Discovery Studio Visualizer 4.1 client [63]. The selected compounds (palmitic acid linolenyl alcohol) were structured using Advanced Chemistry Development/ChemSketch (ACD/ChemSketch) freeware [64]. The generated structures were saved in .mol2 file format, which then was converted to .pdb file format using Open Babel: The Open Source Chemistry Toolbox.

#### 3.6.3. Molecular Docking Analysis

Molecular docking analysis was conducted using the automated docking tool AutoDock 4.2 [65]. Polar hydrogen atoms and Gasteiger partial charges were added to the three-dimensional protein structure. The protein structure was written in .pdbqt file format for further analysis. In this study, the grid size was set at 40 × 40 × 40 points, with 0.375 Å spacing centered on TNF-α, with grid centers x (−13.687), y (71.607), and z (27.002); 40 × 40 × 40 points with 0.375 Å spacing centered on p53-binding protein Mdm-2, with grid centers x (22.407), y (−17.053), and z (−7.329); and 40 × 40 × 40 points with 0.375 Å spacing centered on caspase-3, with grid centers x (27.543), y (22.594), and z (37.217). Lamarckian Genetic Algorithm 4.2 was used in the docking analysis [66] and the protein macromolecules were kept rigid throughout the docking simulation. Genetic algorithm runs were set at 100 and the other parameters for docking analyses were left at default settings. The best protein–compound conformations were chosen from the AutoDock 4.2 scoring function, and they were ranked according to their binding affinities. Discovery Studio Visualizer 4.1 client [63], Chimera 1.14 [67], and LigPlot [68] were used for post-docking analyses.

## 4. Conclusions

The TPC, TFC, antioxidant scavenging activity, antiproliferative activities, and molecular docking of *C. nutans* leaf extracts were determined in this study. The TPC of *C. nutans* leaf extracts was higher than that of TFC. In addition, the CN-Hex fraction had the lowest antioxidant activity and the CN-Crd fraction had the highest antioxidant activity based on the EC_50_ value. The CN-Dcm extract was chosen to study the antiproliferative effect of the leaf extract because it inhibited MCF7 cell growth and was less toxic towards MCF 10A cells. Molecular docking results showed that palmitic acid and linolenyl alcohol from the CN-Dcm fraction could bind with three selected apoptosis-related proteins, and that p53-binding protein Mdm-2 had the highest binding affinity with both compounds. These results suggest that CN-Dcm would be useful for identifying the compounds that best inhibit proliferation of cancer cells and in other applications for determining the apoptosis signaling pathway. For future studies, we would like to isolate and purify the potential active compounds from CN-Dcm fractions. Moreover, it would be worthwhile if we investigated the active compound activity against apoptosis analysis using RNA and protein expression.

## Figures and Tables

**Table 1:** Percentage yield of crude and fraction extracts of *C. nutans* leaves.

Type of Extracts	Percentage Yield (%)
CN-Crd	6.85 ± 0.15 ^a^
CN-Hex	4.10 ± 0.08 ^b^
CN-Dcm	13.73 ± 0.05 ^c^
CN-Chl	2.18 ± 0.24 ^d^
CN-But	20.16 ± 0.34 ^e^
CN-Aqu	37.71 ± 0.09 ^f^

Effect of different solvents on extraction yield. The percentage yield shows significantly different (*p* < 0.05). Different lowercase characters represent significant difference (*p* < 0.05). CN-Crd, crude methanolic extract; CN-Hex, hexane fraction extract; CN-Dcm, dichloromethane fraction extract; CN-Chl, chloroform fraction extract; CN-But, *n*-butanol fraction extract; CN-Aqu, aqueous residue fraction extract.

**Table 2:** The half-maximal effective concentration (EC_50_) of *C. nutans* crude and fraction extracts for 2,2-diphenyl-1-picrylhydrazyl (DPPH) and 2,2’-azino-bis(3-ethylbenzothiazoline-6-sulfonic acid (ABTS) radical scavenging activities.

Type of Extracts	DPPH (µg/mL)	ABTS (µg/mL)
CN-Crd	560.50 ± 2.45	476.30 ± 0.74
CN-Hex	1530.00 ± 3.74	1024.00 ± 4.18
CN-Dcm	1039.00 ± 0.87	937.00 ± 3.84
CN-Chl	796.40 ± 7.21	602.50 ± 1.74
CN-But	837.10 ± 3.14	837.40 ± 9.45
CN-Aqu	744.30 ± 8.45	718.00 ± 1.84
Trolox standard	32.33 ± 2.47	37.74 ± 2.15

Effect of EC_50_ of *C. nutans* crude and fraction extracts. The EC_50_ are significantly different (*p* < 0.05). CN-Crd, crude methanolic extract; CN-Hex, hexane fraction extract; CN-Dcm, dichloromethane fraction extract; CN-Chl, chloroform fraction extract; CN-But, *n*-butanol fraction extract; CN-Aqu, aqueous residue fraction extract.

**Table 3:** Selective index of crude and fraction extracts of *C. nutans* leaf activities.

Selective Index	MCF 10A/MCF7
CN-Crd	0.11
CN-Hex	0.80
CN-Dcm	1.48
CN-Chl	0.85
CN-But	0.78
CN-Aqu	0.4
Tamoxifen	0.49

CN-Crd, crude methanolic extract; CN-Hex, hexane fraction extract; CN-Dcm, dichloromethane fraction extract; CN-Chl, chloroform fraction extract; CN-But, *n*-butanol fraction extract; CN-Aqu, aqueous residue fraction extract.

**Table 4:** Results of the GC-MS analysis of the CN-Dcm fraction of the extract.

Peak	Compound	Retention Time	Molecular Formula	Similarity Index	Peak Percentage (%)
1	Methyl beta-d-glucopyranoside	9.028	C_7_H_14_O_6_	90	0.97
2	Methyl 4-hydroxycinnamate	9.514	C_10_H_10_O_3_	91	3.81
3	Methyl trans-3-hydroxycinnamate	10.077	C_10_H_10_O_3_	94	11.37
4	Methyl palmitate	10.96	C_17_H_34_O_2_	99	1.55
5	Palmitic acid	11.133	C_16_H_32_O_2_	99	23.84
6	10,13-Octadecadienoic acid, methyl ester	11.807	C_19_H_34_O_2_	99	0.92
7	Methyl linolenate	11.842	C_19_H_32_O_2_	99	5.86
8	Phytol	11.891	C_20_H_40_O	80	0.60
9	Methyl stearate	11.926	C_19_H_38_O_2_	99	0.53
10	Linolenyl alcohol	12.023	C_18_H_32_O	95	29.10
11	Octadecanoic acid	12.078	C_18_H_36_O_2_	99	6.15
12	2-(((2-Ethylhexyl)oxy)carbonyl)benzoic acid	14.045	C_16_H_22_O_4_	91	14.51
13	Glyceryl 2-linolenate	15.053	C_21_H_36_O_4_	99	0.44
14	Oleamide	15.706	C_18_H_35_NO	90	0.34

**Table 5:** Lipinski’s rule for major compounds of CN-Dcm, assessed by SwissADME web tool.

Compound	Molecular Weight (Da)	Hydrogen Bond Donor	Hydrogen Bond Acceptor	LogP	Molar Refractivity	Rules Satisfied
Palmitic acid	256.42	1	2	4.19	80.80	5/5
Linolenyl alcohol	264.45	1	1	4.59	88.38	5/5

**Table 6:** Binding energy of major compounds and apoptosis proteins. TNF-α, tumor necrosis factor-α.

Proteins	Binding Energy (kcal/mol)	
	Palmitic Acid	Linolenyl Alcohol
TNF-α	−2.45	−2.99
p53-binding protein Mdm-2	−4.22	−4.56
Caspase-3	−3.56	−3.93

## Supplementary Materials

The following are available online: Table S1: The IC_50_ of *C. nutans* extracts using sulforhodamine B (SRB), 3-(4,5-dimethylthiazol-2-yl)-5-(3-carboxymethoxyphenyl)-2-(4-sulfophenyl)-2*H*-tetrazolium (MTS), and alamar blue assays at 72 h exposure. The antiproliferative effects were evaluated by using MCF7 and MCF 10A.
