# Supplementary material for: Antioxidant Effects, Antiproliferative Effects, and Molecular Docking of Clinacanthus nutans Leaf Extracts
Source: Molecules. 2020 Apr 29;25(9):2067. doi: 10.3390/molecules25092067 (PMC7249086; doi:10.3390/molecules25092067)
Supplement: Supplementary file 1 [file molecules-25-02067-s001.pdf]

Antioxidant, anti-proliferative effects, and molecular docking of *Clinacanthus nutans* leaf extracts

Noor Zafirah Ismail [1], Zaleha Md Toha [1], Musthahimah Muhamad [1], Nik Nur Syazni Nik Mohamed Kamal [1], Nur Nadhirah Mohamad Zain [1] and Hasni Arsad [1,*]

[1]Advanced Medical and Dental Institute, Universiti Sains Malaysia, Bertam, 13200 Kepala Batas, Penang, Malaysia; piecesnzi@gmail.com (N.Z.I); Zaleha.mdtoha@usm.my (Z.M.T); musthahimahmuhamad@gmail.com (M.M); niksyazni@usm.my (N.N.S.N.M.K), nurnadhirah@usm.my (N.N.M.Z); hasniarsad@usm.my (H.A)

[*]Correspondence: hasniarsad@usm.my (H.A); Tel.: +604-5622415 (H.A)

Table S1. The IC_{50} of *C. nutans* extracts using Sulforhodamine B (SRB), 3-(4,5-dimethylthiazol-2-yl)-5-(3-carboxymethoxyphenyl)-2-(4-sulfophenyl)-2H-tetrazolium (MTS) and alamar blue assays at 72 h exposure. The anti-proliferative effects were evaluated by using MCF7 and MCF 10A.

**Type of extracts**	**IC_{50} of *C. nutans* extracts**		
	Alamar blue (µg/mL)	SRB (µg/mL)	MTS (µg/mL)
**MCF7**			
CN-Hex	52.14 ± 1.12	50.34 ± 0.11	50.15 ± 0.75
CN-Dcm	66.47 ± 0.54	65.95 ± 0.14	63.45 ± 0.95
CN-Chl	67.95 ± 1.47	67.52 ± 0.17	62.47 ± 0.47
CN-But	124.41 ± 2.34	111.50 ± 0.20	112.54 ± 1.25
CN-Crd	502.61 ± 5.12	496.50 ± 0.45	475.14 ± 5.14
CN-Aqu	408.67 ± 4.17	398.00 ± 0.24	364.12 ± 4.89
**MCF 10A**			
CN-Hex	37.21 ± 0.87	40.43 ± 1.70	51.13 ± 1.25
CN-Dcm	115.47 ± 1.76	100.20 ± 2.88	100.84 ± 2.49
CN-Chl	56.87 ± 1.24	57.55 ± 0.38	52.14 ± 1.48
CN-But	89.64 ± 1.74	86.50 ± 1.06	89.14 ± 1.47
CN-Crd	52.31 ± 0.84	53.15 ± 0.23	52.78 ± 1.34
CN-Aqu	164.57 ± 2.74	160.40 ± 0.52	16178 ± 3.45

No significant differences between three cytotoxicity assays ($P>0.05$) based on Kruskal-Wallis test analysis, n=3.
